# Redox-Initiated RAFT Emulsion Polymerization-Induced Self-Assembly of *β*-Ketoester Functional Monomers

**DOI:** 10.3390/polym17070870

**Published:** 2025-03-24

**Authors:** Yanfei Wu, Min Han, Xianrong Shen, Qingping Song, Dongdong Liu, Wei Zhang

**Affiliations:** 1 School of Chemical and Environmental Engineering, Anhui Polytechnic University, Wuhu 241000, China; wyf18355047521@163.com (Y.W.); hanmin0456@163.com (M.H.); songqp@ahpu.edu.cn (Q.S.); 2 State and Local Joint Engineering Laboratory for Novel Functional Polymeric Materials, Jiangsu Engineering Laboratory of Novel Functional Polymeric Materials, Suzhou Key Laboratory of Macromolecular Design and Precision Synthesis, College of Chemistry, Chemical Engineering and Materials Science, Soochow University, Suzhou 215123, China

**Keywords:** RAFT polymerization, block copolymers, emulsion polymerization, polymerization-induced self-assembly

## Abstract

Amphiphilic block copolymers are essential for developing advanced polymer nanomaterials with applications in bioimaging, drug delivery, and nanoreactors. In this study, we successfully synthesized functional block copolymer assemblies at high concentrations through redox-initiated reversible addition–fragmentation chain transfer (RAFT) emulsion polymerization of 2-(acetoacetoxy)ethyl methacrylate (AEMA), a *β*-ketoester functional monomer. Utilizing a redox initiation system at 50 °C, we produced poly(poly(ethylene glycol) methyl ether methacrylate)-*b*-PAEMA (PPEGMA_n_-PAEMA_m_). Kinetic studies demonstrated rapid monomer conversion exceeding 95% within 30 min, with distinct polymerization phases driven by micelle formation and monomer depletion. Transmission Electron Microscopy (TEM) and Dynamic Light Scattering (DLS) revealed the formation of diverse morphologies, including worm-like, vesicular structures, and spherical micelles, depending on the macro-CTA molecular weight and monomer concentration. Additionally, post-polymerization modification with aggregation-induced emission (AIE) luminogens, such as 1-(4-aminophenyl)-1,2,2-tristyrene (TPE-NH_2_), resulted in AIE-active polymer assemblies exhibiting strong fluorescence in aqueous dispersions. These AIE-active polymer assemblies also exhibited good biocompatibility. These findings demonstrate the efficacy of redox-initiated RAFT emulsion polymerization in fabricating functional, scalable block copolymer assemblies with potential applications in the field of life sciences.

## 1. Introduction

Amphiphilic block copolymers are widely recognized for their ability to self-assemble in solution, forming a diverse array of nano-objects such as nanospheres, worms, and vesicles with varying compositions and architectures [1]. These nano-objects have attracted significant attention due to their wide-ranging applications in bioimaging, Pickering emulsion, nanoreactors, drug delivery, and biomineralization [2,3,4,5,6,7]. However, conventional polymer solution self-assembly techniques generally operate at low solid content (typically below 1 wt%) and require post-polymerization processing steps, such as pH adjustment or solvent exchange. These limitations hinder the large-scale production of block copolymer nano-objects.

Advancements in living polymerization techniques have greatly facilitated the synthesis of well-defined amphiphilic polymers, thereby significantly enhancing the precision of polymer self-assembly processes [8,9,10,11]. In recent years, a novel technique for preparing polymer nano-objects has emerged from living heterogeneous polymerization technologies, also known as polymerization-induced self-assembly (PISA) [12,13,14,15,16,17,18,19,20]. This technique enables the synthesis of amphiphilic polymers and their in situ self-assembly, promoting the widespread application of polymer nano-objects.

Currently, the most extensively studied PISA methods are based on reversible addition–fragmentation chain transfer (RAFT) polymerization [21,22,23,24,25,26,27,28,29,30,31,32]. In RAFT-mediated PISA, a soluble macromolecular chain transfer agent (macro-CTA) is chain-extended with a monomer to form a block copolymer that self-assembles into nano-objects during the polymerization process. This method enables the preparation of block copolymer nano-objects at high solids contents (10–50% *w*/*w*), making it both scalable and industrially viable. RAFT-mediated PISA can be conducted via RAFT-mediated dispersion polymerization or aqueous emulsion polymerization. The latter has attracted considerable interest due to the use of water as the reaction medium and its compatibility with a wide range of monomers.

Despite these advantages, RAFT-mediated aqueous emulsion polymerization often results in kinetically trapped spherical micelles, making it challenging to achieve higher-order morphologies like worms and vesicles compared to dispersion polymerization [33]. Several research efforts have aimed to address this limitation. Charleux et al. [34] pioneered the aqueous RAFT-mediated emulsion polymerization capable of producing non-spherical nano-objects using poly(acrylic acid-*co*-poly(ethylene glycol) methyl ether acrylate) macro-CTAs with styrene as the monomer. They found that factors such as pH, salt concentration, and stirring speed significantly influenced the resulting morphologies. Hawkett et al. [35] developed a surfactant-free RAFT-mediated emulsion polymerization of styrene, achieving various morphologies including spheres, worm-like structures, and vesicles at high solid contents. Recently, Tan et al. [36] first reported PISA of tert-butyl acrylate (*t*BA) via visible light-initiated RAFT emulsion polymerization, successfully achieving higher-order morphologies (worms and vesicles) of block copolymer assemblies at room temperature. In addition, they [33] reported a PISA system using nonionic, water-soluble macro-CTAs for the redox-initiated RAFT emulsion polymerization of glycidyl methacrylate (GlyMA). A variety of block copolymer assemblies were obtained at relatively low temperatures (25 to 50 °C). They found that increasing the reaction temperature facilitated the formation of higher-order morphologies with narrow molecular weight distribution. They also discovered that the water solubility of the monomer and the *T*_g_ value of the resulting polymer are two key factors influencing morphological evolution under PISA conditions in RAFT emulsion polymerization.

To broaden the scope of RAFT emulsion polymerization, it is essential to develop a wider range of suitable monomers, particularly functional monomers. This advancement will facilitate the synthesis of functional block copolymer assemblies with higher-order morphologies, thereby enhancing their potential for advanced applications. 2-(Acetoacetoxy)ethyl methacrylate (AEMA) is highly valued for its reactive *β*-ketoester groups, which facilitate a wide range of chemical transformations [37,38,39]. These functional groups offer a versatile foundation for post-polymerization modifications, allowing for the incorporation of additional functionalities such as cross-linking sites, coordination sites for metal ions, and reactive handles for further conjugation reactions [40,41,42,43]. This versatility makes AEMA an excellent candidate for designing functional polymers with enhanced properties and diverse applications. Although several studies have reported the preparation of *β*-ketoester functional block copolymer assemblies via RAFT dispersion polymerization of AEMA, the use of emulsion polymerization for this purpose remains rare [44,45,46]. Furthermore, achieving pure worm-like morphologies is challenging when employing RAFT dispersion polymerization of AEMA.

**Scheme 1:**
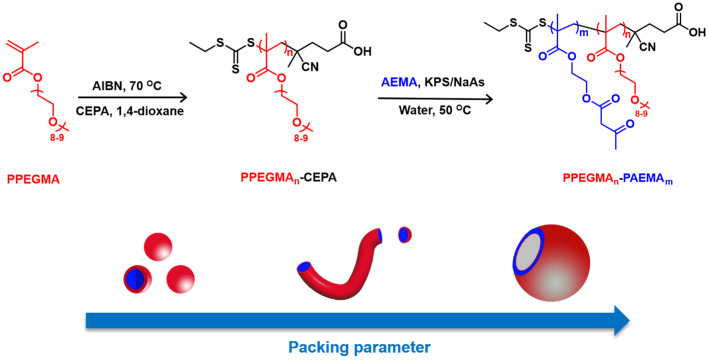
Synthesis of poly(poly(ethylene glycol) methyl ether methacrylate)-poly(2-(acetoacetoxy)ethyl methacrylate) (PPEGMA_n_-PAEMA_m_) diblock copolymer assemblies via redox-initiated RAFT-mediated emulsion polymerization.

In this work, we present a comprehensive study on the Redox-Initiated RAFT emulsion polymerization-induced self-assembly of AEMA (Scheme 1). We aim to synthesize block copolymer assemblies and investigate their self-assembly behavior and morphological evolution. Furthermore, we explore post-polymerization modifications of the *β*-ketoester groups to demonstrate the versatility and potential applications of these functional assemblies. This research contributes to the field by providing new insights into the design and synthesis of functional polymeric nanomaterials with customizable features.

## 2. Materials and Methods

### 2.1. Materials

2-(Acetoacetoxy)ethyl methacrylate (AEMA, Energy Chemical, Shanghai, China) were passed through a column of basic alumina (3A Chem, Anqing, China) prior to storage under refrigeration at 4 °C. Poly(ethylene glycol) methyl ether methacrylate (PEGMA, *M*_n_ = 475 g/mol, Aladdin, Shanghai, China) and hydroquinone (Aladdin, Shanghai, China) were used as received. Azobisisobutyronitrile (AIBN, Energy Chemical, Shanghai, China) was recrystallized from ethanol prior to storage under refrigeration at 4 °C. *L*-Ascorbic acid sodium salt (NaAs) and potassium persulfate (KPS) were purchased from Energy Chemical (Shanghai, China) and Aladdin (Shanghai, China), respectively. KPS was recrystallized from cold water. 4-Cyano-4-(ethylthiocarbonothioylthio) pentanoic acid (CEPA) were synthesized according to a published procedure [47]. Other reagents, including 1-(4-aminophenyl)-1,2,2-tristyrene and 4-(diphenylamino)benzaldehyde were purchased from Energy Chemical (Shanghai, China).

Hela cells were acquired from iCell Bioscience Inc, Shanghai. 3-(4,5-Dimethylthiazol-2-yl)-2,5-diphenyl tetrazolium bromide (MTT) was purchased from Beijing Solarbio Science & Technology Co., Ltd. (Beijing, China). Minimum Essential Medium (MEM, Corning^®^), fetal bovine serum, and antibiotics were purchased from Sigma-Aldrich (Shanghai, China). Trypsin, PBS, and dimethyl sulfoxide (DMSO) were purchased from Aladdin (Shanghai, China).

### 2.2. Synthesis of PPEGMA_n_-CEPA Macro-CTAs

A typical protocol for the synthesis of PPEGMA_12_-CEPA is given below: PEGMA (30.0 g, 63.2 mmol), CEPA (1.11 g, 4.2 mmol), AIBN (0.07 g, 0.43 mmol), 1,3,5-trioxacyclohexane (0.57 g, 6.32 mmol), and 1,4-dioxane (45.0 g) were weighed into a 250 mL round bottom flask and purged with nitrogen for 45 min. The flask was then immersed into a preheated oil bath at 70 °C for 470 min (monomer conversion = 80% as judged by ^1^H NMR spectroscopy). The reaction was then quenched by immersion in ice water and exposure to air. The product was precipitated by adding excess of *n*-hexane (500 mL) and washed several times with additional diethyl ether. The precipitated product was then dried at 45 °C under vacuum overnight. ^1^H NMR measurement indicated a mean degree of polymerization of 12 for this macro-CTA (denoted as PPEGMA_12_-CEPA). Other PPEGMA_n_-CEPA macro-CTAs were synthesized using the same procedure.

### 2.3. Redox-Initiated RAFT Emulsion Polymerization of AEMA

A typical protocol for RAFT-mediated emulsion polymerization of AEMA at 15% *w*/*w* monomer feeding with a target DP of 100 is given as follows: PPEGMA_12_-CEPA (0.28 g, 0.05 mmol), AEMA (1.0 g, 4.67 mmol) were weighed into a 10 mL round bottom flask. Then a certain amount of H_2_O (5.52 g in this case) was added into the flask to dissolve all reagents. The reaction mixture was sealed and degassed with nitrogen for 20 min at 50 °C in a water bath. After the temperature was stabilized, degassed solutions of KPS (84 µL, 50 mg/mL 0.016 mmol) and NaAs (62 µL, 50 mg/mL 0.016 mmol) (the molar ratio of [NaAs]:[KPS]:[PPEGMA_12_-CEPA] = 0.3:0.3:1) were injected in that order via microsyringes. The polymerization was allowed to continue under nitrogen protection for 3 h to ensure full monomer conversion. The polymerization was quenched by exposure to air and the addition of a small amount of hydroquinone. All RAFT emulsion polymerizations were conducted following this protocol to ensure consistency across experiments.

### 2.4. Kinetics of Redox-Initiated RAFT Emulsion Polymerization of PPEGMA_12_-PAEMA_100_

PPEGMA_12_-CEPA (0.70 g, 0.12 mmol), AEMA (2.5 g, 11.7 mmol), and DMF (0.6 mL) were weighed into a 25 mL round bottom flask. Then a certain amount of H_2_O (12.87 g in this case) was added into the flask to dissolve all reagents. The reaction mixture was sealed and degassed with nitrogen for 20 min at 50 °C in a water bath. After the temperature was stabilized, degassed solutions of KPS (525 µL, 20 mg/mL 0.039 mmol) and NaAs (770 µL, 10 mg/mL 0.039 mmol) (the molar ratio of [NaAs]:[KPS]:[PPEGMA_12_-CEPA] = 0.3:0.3:1) were injected in that order via microsyringes. Samples were withdrawn at predetermined time intervals by syringes under nitrogen, and the polymerization was quenched by exposure to air and the addition of a small amount of hydroquinone. The samples were then analyzed by ^1^H NMR spectroscopy.

### 2.5. Preparation of AIE-Active Polymer Assemblies

A typical procedure for preparing amino-functionalized AIE-active polymer assemblies is described as follows: PPEGMA_12_-PAEMA_150_ polymer assemblies (0.110 g, prepared at a monomer concentration of 20% *w*/*w*) were added to a 5 mL glass vial containing water (1.8 g) under stirring. In a separate vial, TPE-NH_2_ (0.005 g) was dissolved in DMF (0.5 mL). This TPE-NH_2_ solution was then added dropwise to the polymer solution under continuous stirring. The resulting mixture was stirred for 24 h to ensure the formation of stable AIE-active polymer assemblies.

### 2.6. In Vitro Cytotoxicity Assay

The viability of cells exposed to AIE-active polymer assemblies was assessed using the MTT method. Typically, Hela cells were seeded in 96-well plates at a density of 6 × 10^3^ cells per well and cultured overnight with 100 μL of MEM medium. Afterward, varying concentrations of AIE-active polymer assemblies were added, and the cells were incubated for an additional 24 h under 5% CO_2_ at 37 °C. Following incubation, 100 μL of MTT solution (0.5 mg/mL in PBS) was added to each well, and the cells were cultured for 4 h. Then, 100 μL of DMSO was added to replace the culture medium. Absorbance was measured at 570 nm using a microplate reader (SparK 10M, Tecan, Männedorf, Switzerland).

### 2.7. Characterization

^1^H Nuclear magnetic resonance (NMR) spectroscopy; NMR spectra were recorded in CDCl_3_ or DMSO-*d*_6_ using a Bruker Avance NEO 500 MHz NMR spectrometer (Bruker, Billerica, MA, USA) at a temperature of 25 °C. This technique was employed to confirm the chemical structure and purity of the synthesized polymers and to determine the monomer conversion rate during the polymerization reaction.

Transmission electron microscopy (TEM): The obtained polymer dispersions were diluted 50-fold with water. A drop of the solution was placed on a copper grid for 3 min and then blotted with filter paper to remove excess liquid. Following this, a drop of uranyl acetate solution (0.5 wt%) was soaked on the same copper grid for 3 min, and then blotted with filter paper to remove excess strain. TEM observations were carried out on a Hitachi 7800 instrument (Hitachi, Tokyo, Japan) operated at 100 kV. This method was used to observe the morphology of the polymer assemblies.

Gel permeation chromatography (GPC): The molecular weight and polydispersity of the polymers were measured by gel permeation chromatography (GPC) at 35 °C using a Waters 2414 GPC instrument (Waters, Milford, CT, USA)with dimethylformamide (THF) as the mobile phase and Waters styragel HR3 and HR4 columns. The flow rate of THF was 1.0 mL/min. Linear polymethacrylate polymers with narrow molecular weight distributions were used as the standards to calibrate apparatus. GPC was utilized to determine the relative molecular weight and polydispersity of the polymers.

Dynamic light scattering (DLS): The intensity-average hydrodynamic diameters of the copolymer dispersions were determined using a Malvern Panalytical Zetasizer Pro Zeta potential and molecular weight analyzer (Malvern Panalytical, Malvern, UK). Dilute aqueous dispersions were analyzed using disposable cuvettes and all data were averaged over three consecutive runs. DLS was used to measure the hydrodynamic diameter of the polymer assemblies.

Differential scanning calorimetry (DSC): DSC was performed using a differential scanning calorimetric (DSC25 TA Instruments, TA, Newcastle, WA, USA) instrument under a stream of nitrogen. The sample was weighed accurately (5–10 mg). An empty aluminum pan was used as the reference. The standard heating rate used was 10 °C/min, and the temperature range of the scan was from −50 to 150 °C. DSC was used to determine the glass transition temperature of the polymers.

Fluorescence spectroscopy: Fluorescence spectra of aqueous solutions of fluorescent polymers were measured in a JASCO FP-8550 fluorescence spectrophotometer (JASCO, Tokyo, Japan). Test parameters: slit width of 2.5 nm, excitation wavelength of 254 and 350 nm, and scanning speed of 100 nm/min. Fluorescence spectroscopy was used to measure the emission spectra of the aggregation-induced emission (AIE)-active polymer assemblies.

## 3. Results and Discussion

### 3.1. Synthesis of PPEGMA_n_-CEPA Macro-CTAs

**Figure 1:**
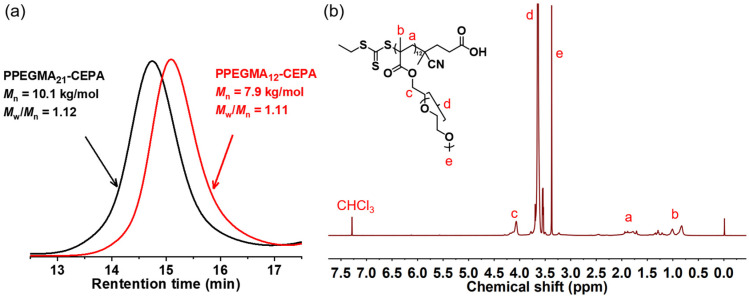
(**a**) THF GPC curves of PPEGMA_n_-CEPA macro-CTAs; (**b**) ^1^H NMR spectra of PPEGMA_12_-CEPA in CDCl_3_.

Brush polymers derived from poly(ethylene glycol) methyl ether methacrylate (PEGMA) are known for their highly water solubility, biocompatibility and antifouling characteristics. These attributes make PEGMA-based polymers valuable stabilizers in mechanistic studies of aqueous PISA [48]. Here, we synthesized PPEGMA_n_-CEPA macro-CTAs via RAFT solution polymerization of PEGMA. In this process, 4-Cyano-4-(ethylthiocarbonothioylthio) pentanoic acid (CEPA) was employed as the RAFT agent, and AIBN served as the thermal initiator. The degree of polymerization (DP) of the resulting PPEGMA polymer was determined based on monomer conversion. Accordingly, two PPEGMA-based macro-CTAs with DPs of 12 and 21 were synthesized, designated as PPEGMA_12_-CEPA and PPEGMA_21_-CEPA, respectively. Tetrahydrofuran (THF) GPC confirmed that both macro-CTAs exhibited narrow molecular weight distribution, as shown in Figure 1a. Specifically, PPEGMA_12_-CEPA had a number-average molecular weight (*M*_n_) of 7.9 kg/mol and a dispersity (*M*_w_/*M*_n_) of 1.11, while PPEGMA_21_-CEPA showed an *M*_n_ of 10.1 kg/mol with *M*_w_/*M*_n_ = 1.12. In addition, PPEGMA_12_-CEPA was further characterized by ^1^H NMR in CDCl_3_, as shown in Figure 1b. From the spectrum, it is evident that polymer peaks emerge at 0.7–1.1 ppm (b) and 1.7–2.0 ppm (a), corresponding to polymerized methacrylate-type monomers. Peak c (4.0–4.5 ppm) corresponds to the methylene protons adjacent to the ester groups in the PPEGMA repeating units. Peak d (3.5–4.0 ppm) represents the methylene protons of the PEG segments in the polymer backbone. Peak e (3.3–3.5 ppm) is assigned to the terminal methyl protons adjacent to the ether bonds within the PPEGMA repeating units. This finding is consistent with observations reported in the relevant literature [44,49].

### 3.2. Kinetic Process of Redox-Initiated RAFT Emulsion Polymerization of AEMA

The initial experiment of aqueous emulsion PISA was carried out in water at 70 °C using KPS/NaAs as the initiator and PPEGMA_12_-CEPA as the macro-CTA. However, the reaction became unstable and only aggregates were obtained. This may be ascribed to the reduced aqueous solubility of PEG chains at this high temperature [50]. Subsequently, leveraging the broad temperature operability of redox-initiated polymerization, we performed the aqueous emulsion PISA of AEMA at 50 °C, as shown in Scheme 1.

To further clarify the process of RAFT emulsion polymerization, kinetic studies were conducted using a custom-built device that maintains a nitrogen atmosphere (Figure S1). Samples were extracted at different time intervals and analyzed by ^1^H NMR spectroscopy to monitor the polymerization progress. In this experiment, a monomer concentration of 15% *w*/*w* was employed, utilizing PPEGMA_12_-CEPA as the macro-CTA, with a target DP of 100 for the hydrophobic segments. The ^1^H NMR spectra of aliquots withdrawn at different times are presented in Figure S2. It is evident that as the polymerization time increases, the vinyl signals decrease significantly, indicating the rapid consumption of the AEMA.

**Figure 2:**
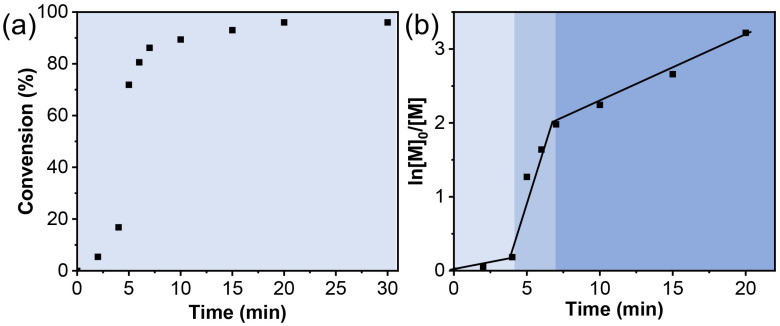
Kinetics of polymerization of AEMA via RAFT-mediated emulsion polymerization (monomer concentration of 15% *w*/*w*, target composition of PPEGMA_12_-PAEMA_100_) at 50 °C (**a**) conversion vs. time plot and (**b**) semi-logarithmic plot of the conversion.

Figure 2a illustrates the evolution of monomer conversion over the reaction time, demonstrating that high monomer conversion (>95%) was achieved within 30 min. The semilogarithmic plots in Figure 2b reveal three distinct phases of the polymerization process. The initial phase (0–4 min) corresponds to the formation of amphiphilic micelles as the amphiphilic propagating radicals self-assemble in water. During the second phase, hydrophobic AEMA monomers are gradually incorporated into the micelles, increasing the local monomer concentration and causing a sharp rise in the polymerization rate. This increase can be attributed to the onset of nucleation of block copolymers, as observed in time-resolved small-angle X-ray scattering (SAXS) investigations by Armes’ group [51,52]. The primary polymerization locus is within the monomer-swollen micelles. In the final phase, as polymerization continues, the monomers are depleted, monomer droplets disappear, and the polymerization rate decreases. These observations suggest that the formation and growth of micelles play a crucial role in the RAFT emulsion polymerization kinetics. The initial self-assembly of amphiphilic radicals facilitates the localized concentration of monomers within micelles, thereby enhancing the polymerization rate during the acceleration stage. As monomer availability diminishes, the system transitions into the deceleration stage, characterized by a reduced polymerization rate. This kinetic process exhibits the characteristics of typical emulsion polymerization and is similar to the results reported in the literature on RAFT emulsion polymerization [53].

### 3.3. Morphological Evolution of Block Copolymer Assemblies in Redox-Initiated RAFT Emulsion Polymerization

**Figure 3:**
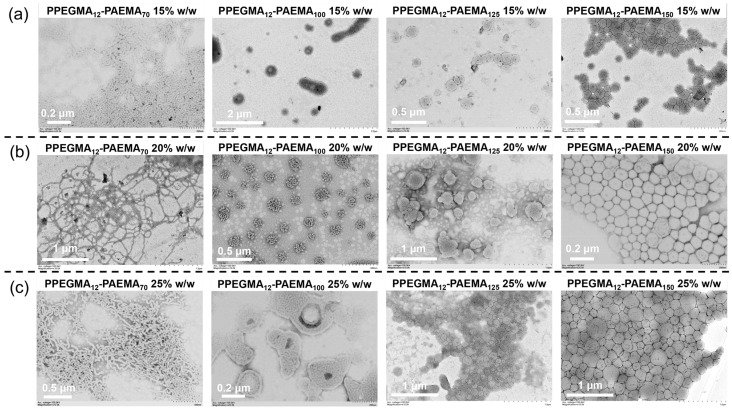
TEM images of PPEGMA_12_-PAEMA_m_ diblock copolymer assemblies prepared by RAFT emulsion polymerization of AEMA at different monomer concentrations. (**a**) 15% *w*/*w*; (**b**) 20% *w*/*w*; (**c**) 25% *w*/*w*.

The morphology of diblock copolymer assemblies prepared through PISA primarily depends on the relative volume fraction of the two blocks, known as the packing parameter (*P*) [54]. In RAFT-PISA, *P* can be tuned by adjusting the DP of the core-forming block and the molecular weight of the macro-CTA. To explore this, we first examined the effect of varying the DP of PAEMA and the monomer concentration on the morphologies of block copolymer assemblies prepared by redox-initiated RAFT emulsion polymerization. Using PPEGMA_12_-CEPA as the macro-CTA, and adjusting both the monomer concentration and the DP of the PAEMA segments, we prepared a series of block copolymer assemblies. These were characterized by TEM, as shown in Figure 3. The TEM images reveal that both pure worm-like and vesicular morphologies were successfully synthesized. Notably, at a PAEMA DP of 100 or 125, a transitional morphology from worm-like to vesicular structures was observed. This transition is driven by changes in the polymerization conditions and the relative volume fractions of the hydrophilic and hydrophobic blocks. Similar to RAFT aqueous dispersion polymerization [21], worm-like micelles initially aggregate and then fuse to form vesicles. However, in the dispersion polymerization of AEMA, the formation of worm-like micelles is typically challenging to observe [44,45].

We also performed GPC characterization on several synthesized PPEGMA_12_-PAEMA_m_ block copolymers, as shown in Figure S3. The GPC results indicate that the synthesized block copolymers exhibit a broad molecular weight distribution, which may be attributed to the formation of branched polymers during the polymerization process. This finding is consistent with previous reports from An et al. [44]. The broad distribution arises because the methylene group distal to the double bond in AEMA is electron-withdrawing due to the influence of the two nearby carbonyl groups, making it prone to dehydrogenation reactions that generate radicals. These radicals can be stabilized through resonance with the dual carbonyl groups on the AEMA units. However, these stabilized radicals may undergo branching side reactions, leading to the production of polymers with slight branching degrees. Importantly, these side reactions do not result in the cross-linking of the block copolymer assemblies. This issue can be mitigated by copolymerizing AEMA with another monomer, which reduces the labile hydrogens in the polymer, minimizes chain transfer to the monomer, and thus improves the dispersity of the block copolymers [45].

Monomer concentration plays a pivotal role in determining the morphologies of block copolymer assemblies in RAFT-mediated dispersion polymerization. Typically, elevated monomer concentrations promote the development of higher-order morphologies, as the monomer functions as a co-solvent for the growing polymer segments [55,56,57]. To assess the influence of monomer concentration on RAFT-mediated emulsion polymerization, we constructed a morphological phase diagram for PPEGMA_12_-PAEMA_m_ diblock copolymer assemblies, as depicted in Figure S4. Contrary to the trends observed in RAFT dispersion polymerization, varying the monomer concentration in RAFT emulsion polymerization did not result in significant changes to the morphological phase diagram. This observation aligns with the findings reported by Tan et al. [33]. The underlying reason for this behavior lies in the fundamental mechanism of RAFT emulsion polymerization. Unlike dispersion polymerization, the monomer exists as separate droplets due to its low solubility in water. As the monomer is consumed within the micelles, it must be gradually transported from these droplets to the micellar cores. Therefore, within the concentration range studied, the overall monomer concentration has a limited effect on the formation of block copolymer assemblies in RAFT emulsion polymerization of AEMA.

**Figure 4:**
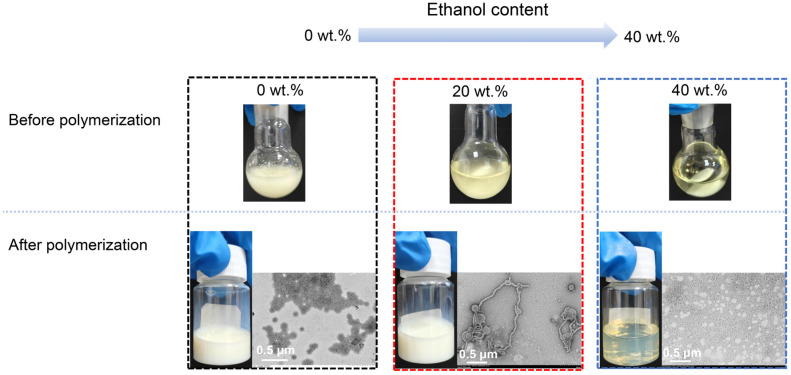
Digital images of reaction mixtures for heterogeneous RAFT polymerization of AEMA (15% *w*/*w*, target DP of 150) mediated by PPEGMA_12_-CEPA in a water/ethanol mixture with different ethanol contents, along with TEM images taken after polymerization.

In RAFT-PISA, the composition of the reaction solvent plays a crucial role in the morphological evolution of block copolymer assemblies. To investigate this, we introduced ethanol into the emulsion polymerization and explored its effect on the morphological evolution of AEMA in the RAFT-PISA process. The monomer concentration was set to 15% *w*/*w*, with the polymer composition being PPEGMA_12_-PAEMA_150_. As shown in Figure 4, before polymerization, a turbid solution was observed due to the presence of AEMA droplets in the aqueous phase. After redox polymerization, a milky white solution formed, indicating the creation of polymer assemblies. Ethanol is a good solvent for AEMA and is miscible with water. The addition of ethanol to the reaction solvent induces a transition from RAFT emulsion polymerization to RAFT dispersion polymerization. At an ethanol/water ratio of 20/80 (*w*/*w*), a portion of AEMA dissolved in the reaction medium, although a turbid solution still appeared before polymerization (Figure 4). Under these conditions, a higher concentration of monomer dissolved in the reaction medium, with the remaining monomer existing in droplet form. At this point, the heterogeneous polymerization proceeds via a mixed mechanism of RAFT emulsion polymerization and RAFT dispersion polymerization. Following redox initiation, the solution turned milky white, indicating the formation of block copolymer assemblies. When the ethanol/water ratio increased to 40/60 (*w*/*w*), AEMA was completely dissolved in the mixed solvent, and an optically transparent solution was observed before polymerization (Figure 4). After redox initiation, a blue solution was formed, signaling that AEMA polymerized through RAFT dispersion polymerization. TEM characterization of the polymerized samples revealed that, without ethanol, the block copolymer assemblies formed purely vesicular morphologies. When the ethanol content increased to 20%, a mixed morphology of worms and vesicles was observed. With an ethanol content of 40%, the morphology transitioned into spherical micelles (Figure 4).

This study demonstrates that increasing ethanol content enhances the solubility of the monomer in the reaction medium, leading to a decrease in the local monomer concentration within the swollen micelles. Additionally, higher ethanol concentrations raise the critical DP of the core-forming block, decreasing the available monomer for polymerization after micellization. Therefore, the transition from RAFT emulsion polymerization to RAFT dispersion polymerization favors the formation of lower-order morphologies, such as spheres. This result is similar to that reported by Tan et al. [58].

**Figure 5:**
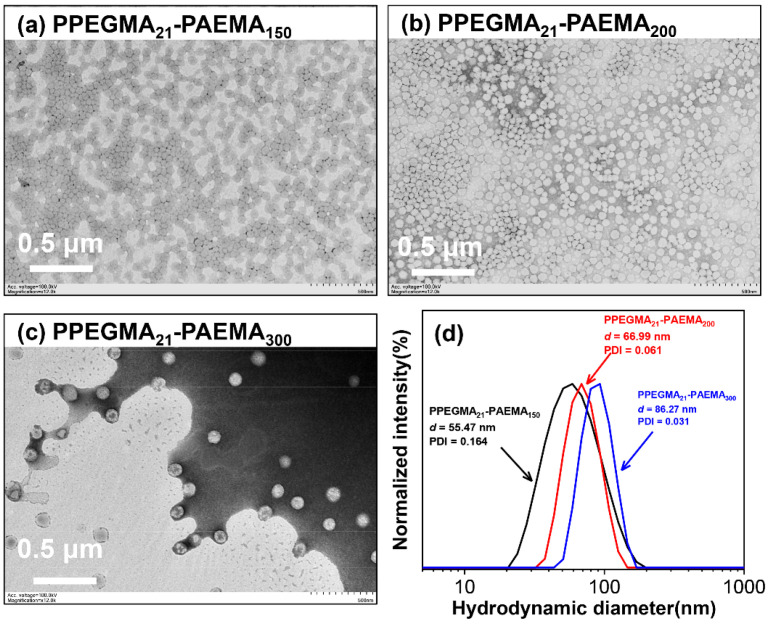
(**a**–**c**) TEM images of PPEGMA_21_-PAEMA_m_ diblock copolymer assemblies prepared by RAFT emulsion polymerization of AEMA (20% *w*/*w*), (**d**) DLS curve corresponding to the polymer assemblies shown in (**a**–**c**).

**Figure 6:**
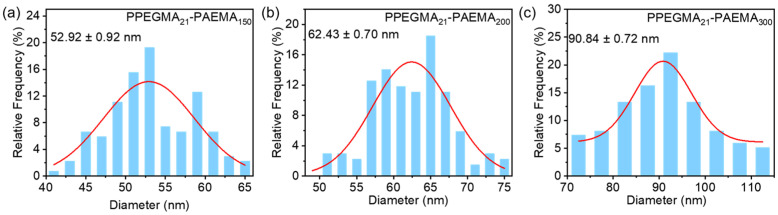
The histogram displays the micelle size distribution obtained from the TEM images of the PPEGMA_21_-PAEMA_m_ (m = 150, 200, 300), with Gaussian fitting (red line) used to determine the average diameter. (**a**) PPEGMA_21_-PAEMA_150_; (**b**) PPEGMA_21_-PAEMA_200_; (**c**) PPEGMA_21_-PAEMA_300._

Although spherical micelles were not observed with PPEGMA_12_-CEPA, we sought to achieve the preparation of spherical micelles by using the higher molecular weight PPEGMA_21_-CEPA to control the emulsion polymerization of AEMA. As shown in Figure 5a–c, at a monomer concentration of 20% *w*/*w*, spherical micelles were formed as the target DP of the PAEMA segment increased from 150 to 300. DLS was used to characterize these spherical micelles, with the results presented in Figure 5d. Specifically, as the DP of the PAEMA block increased from 150 to 200 and 300, the average hydrodynamic diameter grew from 55.47 nm to 66.99 nm and 86.27 nm, respectively. Additionally, Image Pro Plus 6.0 software was used to analyze the micelle size from the TEM images, applying Gaussian fitting (red line) to obtain the mean diameter (Figure 6). As shown in the figure, the micelle size increases gradually with the increasing DP of the PAEMA segment. The average micelle sizes for PAEMA_150_, PAEMA_200_, and PAEMA_300_ were 52.92 ± 0.92 nm, 62.43 ± 0.70 nm, and 90.84 ± 0.72 nm, respectively.

Compared to the average micelle size measured by DLS, the sizes obtained from TEM are generally smaller. Theoretically, this discrepancy is commonly observed because TEM provides a projection of the micelle’s size, which may underestimate the actual size due to the drying and staining processes involved in sample preparation. In contrast, DLS measures the hydrodynamic diameter in solution, which includes the solvation shell around the particles, leading to larger size measurements. This difference underscores the importance of using multiple characterization techniques to gain a more comprehensive understanding of the micelle size and morphology. However, the size of the PAEMA_300_ micelles calculated from the TEM images is larger than that measured by DLS, and we attribute this discrepancy to measurement errors. Several factors could explain this difference. First, analyzing the micelle size from TEM images using software has inherent limitations, including the subjectivity involved in selecting the images and the lack of a holistic approach. Additionally, manual measurements using software can introduce errors, especially since some micelles are not perfectly spherical. Despite these potential issues, the observed trend remains consistent, regardless of whether measurements are taken by DLS or TEM. Specifically, as the length of the PAEMA segment increases, the size of the PPEGMA_21_-PAEMA_m_ diblock polymer micelles gradually increases.

**Figure 7:**
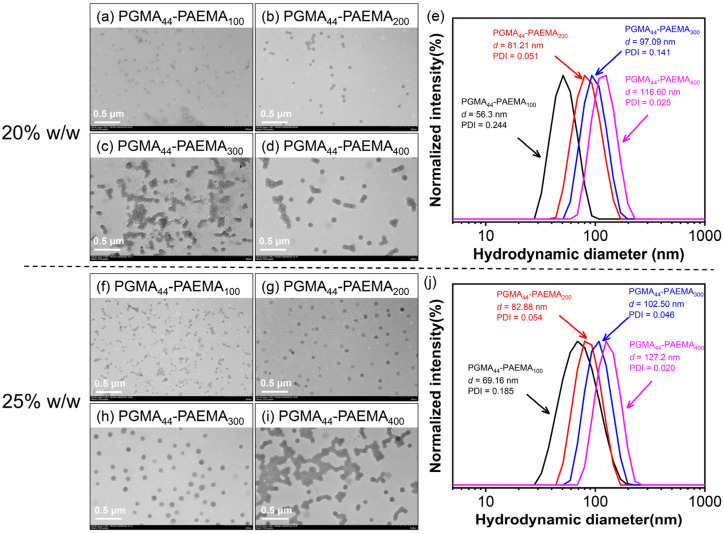
(**a**–**d**) TEM images of PGMA_44_-PAEMA_m_ diblock copolymer assemblies prepared by RAFT emulsion polymerization of AEMA (20% *w*/*w*), (**e**) DLS curve corresponding to the polymer assemblies shown in (**a**–**d**), (**f**–**i**) TEM images of PGMA_44_-PAEMA_m_ diblock copolymer assemblies prepared by RAFT emulsion polymerization of AEMA (25% *w*/*w*), (**j**) DLS curve corresponding to the polymer assemblies shown in (**f**–**i**).

To demonstrate the reproducibility of this redox-initiated PISA system, we also tested a poly(glycerol monomethacrylate) (PGMA)-based macro-CTA to control the emulsion polymerization of AEMA (Figure S6). PGMA, known for its good hydrophilicity, is commonly used in RAFT aqueous dispersion polymerization [59,60]. By varying the monomer concentration and the DP of the PAEMA segment, we prepared a series of PGMA-PAEMA diblock copolymer assemblies. TEM analysis revealed that all these assemblies formed spherical micelles (Figure 7). Additionally, DLS characterization showed that as the DP of the PAEMA segment increased, the size of the spherical micelles also increased (Figure 7e,j). Although PGMA-PAEMA diblock copolymer assemblies with higher-order morphologies (such as worms and vesicles) were not achieved, this result confirms the reproducibility of redox-initiated emulsion polymerization of AEMA. It is worth noting that using a PGMA-based macro-CTA with a smaller molecular weight could potentially facilitate further morphological evolution in RAFT emulsion polymerization of AEMA, though this is beyond the scope of the current study.

### 3.4. Preparation of AIE-Active Polymer Assemblies

In recent years, fluorescent polymer nanoparticles have attracted significant attention from the scientific community due to their extensive applications in optics, electronics, memory systems, and biotechnology [61,62]. In 2001, Tang and colleagues discovered an unusual anti-aggregation-caused quenching (ACQ) photophysical phenomenon known as aggregation-induced emission (AIE) [63]. Since then, AIE-active polymer materials have stimulated intense research interest owing to their fundamental importance and promising practical applications, with the mechanisms and technological applications of AIE materials continuously evolving and improving [64,65,66]. Among the methods for constructing AIE-active polymer materials, post-polymerization modification is a useful strategy to functionalize polymers and endow them with new properties for broad applications [67,68]. The copolymer backbone contains reactive moieties that can be directly functionalized via precursor functional groups.

**Figure 8:**
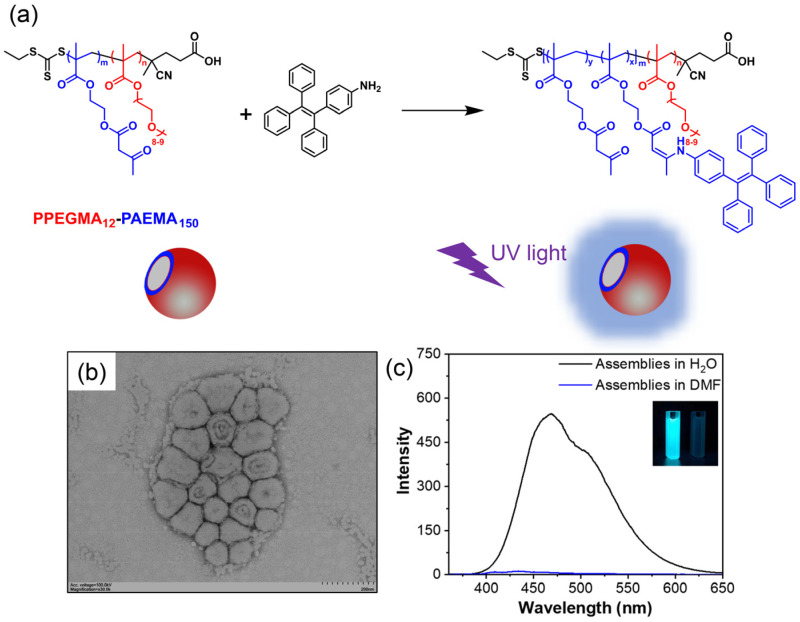
(**a**) Schematic illustration of the post-polymerization modification strategy of PPGMA_12_-PAEMA_150_ block copolymer assemblies prepared via RAFT emulsion polymerization for the fabrication of the AIE-active polymer assemblies. (**b**) TEM images of the AIE polymer nanoparticles prepared using the strategy shown in (**a**). (**c**) FL spectra of the AIE polymer nanoparticles. in DMF and in water. Concentration of particles was 80 μg mL^–1^ for both samples. E_x_ = 350 nm, and the slit width is 2.5 nm. Inset are the optical and fluorescent photographs of the AIE-active polymer assemblies.

As previously noted, *β*-ketoester groups are capable of reacting efficiently with a range of functional groups, including amines and aldehydes. Leveraging this property, we performed post-polymerization modifications on *β*-ketoester functional PPEGMA_12_-PAEMA_m_ diblock copolymer assemblies to produce aggregation-induced emission (AIE) polymer nanoparticles. Specifically, amino-functionalized tetraphenylethylene (TPE-NH_2_) was reacted at room temperature with vesicle-type assemblies (Figure 8a). TEM images confirmed that the modified assemblies preserved their vesicular structure (Figure 8b). To examine the AIE effect, we compared the fluorescence spectra of the TPE-modified block copolymer assemblies in dimethylformamide (DMF) solution and in aqueous dispersion. As shown in Figure 8c, the DMF solution of the TPE-modified assemblies displayed negligible fluorescence at 470 nm, whereas the aqueous dispersions exhibited a strong fluorescence peak at 470 nm. This substantial difference indicates the presence of the AIE effect, which stems from restricted intramolecular rotation of TPE units during aggregation. Moreover, aldehyde-functionalized AIE molecules were also used to modify the assemblies, successfully yielding AIE-active polymer structures (Figure S8).

**Figure 9:**
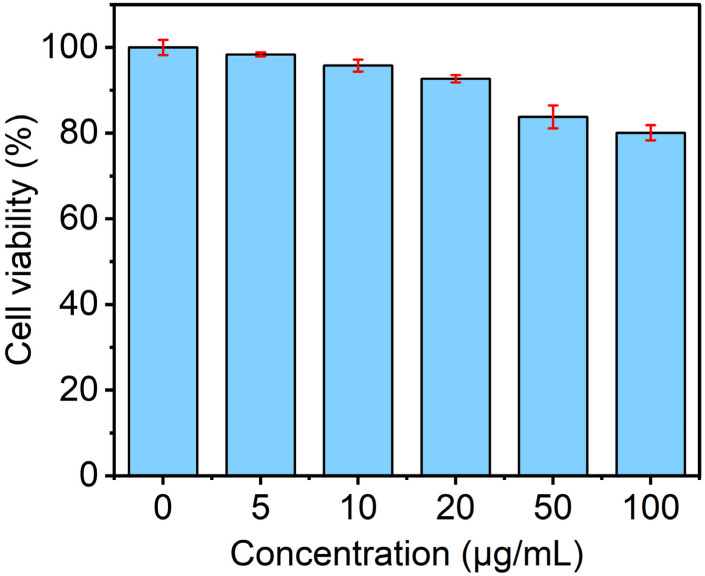
In vitro cytotoxicity of TPE-modified block copolymer assemblies against Hela cells at different concentrations, measured using the MTT assay.

To further explore the potential applications of AIE-active polymer assemblies in biosciences, we assessed their biocompatibility. In this study, the MTT assay was used to evaluate the cytotoxicity of TPE-modified block copolymer assemblies by measuring the viability of HeLa cells. The absorbance was directly proportional to the number of living cells, reflecting the content of amylase in viable cells. Changes in absorbance were calculated by comparing the treated group to the untreated group (which was not cultured in medium containing TPE-modified block copolymer assemblies), thus indicating cell viability. As shown in Figure 9, when the concentration of TPE-modified block copolymer assemblies was below 20 µg/mL, the cell viability of HeLa cells remained nearly unchanged. Even at concentrations as high as 100 µg/mL, cell viability was still above 80% after 24 h of incubation with TPE-modified block copolymer assemblies. These results suggest that the toxicity of the AIE-active polymer assemblies is negligible at concentrations lower than 20 µg/mL. This result demonstrates the multifunctional nature of *β*-ketoester polymer assemblies, which can be used to fabricate fluorescent polymer nanomaterials with good biocompatibility. Moving forward, we plan to explore their potential applications in drug delivery, taking advantage of their modifiability.

## 4. Conclusions

This study successfully synthesized *β*-ketoester functional block copolymer assemblies via redox-initiated RAFT emulsion polymerization. Kinetic studies revealed rapid monomer conversion exceeding 95% within 30 min, characterized by distinct phases of micelle formation, accelerated polymerization, and deceleration due to monomer depletion. The morphology of the diblock copolymer assemblies was controlled by adjusting the DP of the PAEMA block and the monomer concentration, leading to the formation of diverse morphologies, including worm-like structures and vesicles, as characterized by TEM. Unlike RAFT dispersion polymerization, where monomer concentration significantly influences morphology, the results from RAFT emulsion polymerization showed minimal impact from varying monomer concentrations. Furthermore, we studied the effect of solvent composition on the morphology of the diblock copolymer assemblies. The results indicated that increasing the ethanol content led to a change in morphology. When the ethanol content reached 40 wt%, the assemblies transitioned from a vesicular morphology to spherical micelles, at which point the polymerization switched to RAFT dispersion polymerization).

By using the higher molecular weight PPEGMA_21_-CEPA to control the RAFT emulsion polymerization of AEMA, spherical micelles were formed. DLS characterization showed that as the DP of the PAEMA block increased, the size of the assemblies also increased. Specifically, as the DP of the PAEMA block increased from 150 to 200 and 300, the average hydrodynamic diameter increased from 55.47 nm to 66.99 nm and 86.27 nm, respectively. Although the particle sizes obtained from TEM images differed from the DLS measurements, the overall trend remained consistent. Additionally, we synthesized PGMA-PAEMA diblock copolymer assemblies using this method, demonstrating the reproducibility of the redox-initiated RAFT emulsion polymerization.

Finally, successful post-polymerization modification with AIE luminogens such as TPE-NH_2_ demonstrated the potential for creating fluorescent nanostructures without compromising structural integrity. We also assessed the biocompatibility of the assemblies, and the results indicated that the TPE-modified block copolymer assemblies exhibited negligible cytotoxicity when the concentration was below 20 µg/mL.

Future research should explore the incorporation of additional functional monomers to impart stimuli responsiveness or targeting capabilities, further enhancing the applicability of these block copolymer assemblies. Additionally, investigating the scalability and industrial viability of redox-initiated RAFT emulsion polymerization could facilitate the transition from laboratory synthesis to practical applications, expanding the utility of RAFT-mediated PISA in various advanced material systems. Overall, this work advances the controlled synthesis of functional polymer nano-objects, providing a robust foundation for future innovations in nanotechnology, biomedical engineering, and materials science.

## Data Availability

The original contributions presented in the study are included in the article/supplementary material, further inquiries can be directed to the corresponding authors.

## Supplementary Materials

The following supporting information can be downloaded at: www.mdpi.com/article/10.3390/polym17070870/s1, Table S1: Formulation for preparing PPEGMA_n_-PAEMA_m_ block copolymer assemblies via redox-initiated RAFT emulsion polymerization. Figure S1: Schematic diagram of the custom-built device for the redox-initiated RAFT emulsion polymerization kinetics of AEMA (nitrogen flow through the tube during operation). Figure S2: ^1^H NMR spectra of aliquots withdrawn during RAFT emulsion polymerization of AEMA of 15% *w*/*w*, target composition of PPEPMA_12_-PAEMA_100_ at 50 °C. A small amount of DMF was used as the internal standard. Figure S3: THF GPC traces of diblock copolymers formed by RAFT emulsion polymerization of AEMA (20% *w*/*w*). Figure S4: Morphological phase diagram constructed for PPEGMA_12_-PAEMA_m_ diblock copolymer assemblies prepared by RAFT emulsion polymerization of AEMA. Figure S5: DSC curve of the block copolymer PPEGMA_12_-PAEMA_100_ obtained by RAFT emulsion polymerization of AEMA. Figure S6: Synthesis of poly(glycerol monomethacrylate)-poly(2-(acetoacetoxy) ethyl methacrylate) (PGMA_44_-PAEMA_m_) diblock copolymer assemblies via redox-initiated RAFT-mediated emulsion polymerization. Figure S7: ^1^H NMR spectra of AIE-polymer assemblies in CDCl_3_, prepared via post-polymerization modification. Figure S8: Schematic illustration of the preparation of AIE polymer assemblies via post-polymerization modification with aldehyde-based AIE molecules, along with the fluorescence emission spectra of the AIE polymer assemblies.

## Abbreviations

The following abbreviations are used in this manuscript:

PISA	polymerization-induced self-assembly
RAFT	reversible addition–fragmentation chain transfer
Macro-CTA	macromolecular chain transfer agent
DP	degree of polymerization
*t*BA	tert-butyl acrylate
GlyMA	glycidyl methacrylate
AEMA	2-(acetoacetoxy)ethyl methacrylate
PPEGMA	poly(poly(ethylene glycol) methyl ether methacrylate)
AIBN	azobisisobutyronitrile
NaAs	*L*-Ascorbic acid sodium salt
KPS	potassium persulfate
CEPA	4-cyano-4-(ethylthiocarbonothioylthio) pentanoic acid
NMR	nuclear magnetic resonance
TEM	transmission electron microscopy
GPC	gel permeation chromatography
DSC	dynamic light scattering
ACQ	aggregation-caused quenching
AIE	aggregation-induced emission
TPE	tetraphenylethylene
DMF	dimethylformamide
THF	tetrahydrofuran
