# Supplementary material for: Redox-Initiated RAFT Emulsion Polymerization-Induced Self-Assembly of β-Ketoester Functional Monomers
Source: Polymers (Basel). 2025 Mar 24;17(7):870. doi: 10.3390/polym17070870 (PMC11990964; doi:10.3390/polym17070870)
Supplement: Supplementary file 1 [file polymers-17-00870-s001.zip › polymers-3446640-supplementary.pdf]

Supporting Information for

Redox-Initiated RAFT Emulsion Polymerization-Induced Self-Assembly of *β*-Ketoester Functional Monomers

Yanfei Wu[1,†], Min Han[1,†], Xianrong Shen[1,*], Qingping Song[1], Dongdong Liu[1,*] and Wei Zhang[2,*]

[1]. School of Chemical and Environmental Engineering, Anhui Polytechnic University, Wuhu 241000, China.
[2]. State and Local Joint Engineering Laboratory for Novel Functional Polymeric Materials, Jiangsu Engineering Laboratory of Novel Functional Polymeric Materials, Suzhou Key Laboratory of Macromolecular Design and Precision Synthesis, College of Chemistry, Chemical Engineering and Materials Science, Soochow University, Suzhou 215123, China
[†] These authors contributed equally to this work.

*Corresponding authors: shenxr@ahpu.edu.cn; liudongdong@ahpu.edu.cn; weizhang@suda.edu.cn

Supporting Figures.

Table S1. Formulation for preparing $PPEGMA_n$-$PAEMA_m$ block copolymer assembiles via redox-initiated RAFT emulsion polymerization.

Monomer Concentration (% w/w)	Target composition	AEMA (g)	Macro-CTA (g)	H_2O (g)	KPS (50 mg/mL in H_2O)	NaAs (50 mg/mL in H_2O)
15	$PPEGMA_{12}$-$PAEMA_{70}$	0.50	0.1988	2.729	60 μL	44 μL
	$PPEGMA_{12}$-$PAEMA_{100}$	1.00	0.2784	5.521	84 μL	62 μL
	$PPEGMA_{12}$-$PAEMA_{125}$	1.50	0.3340	8.326	100 μL	74 μL
	$PPEGMA_{12}$-$PAEMA_{150}$	1.20	0.2227	6.682	68 μL	50 μL
20	$PPEGMA_{12}$-$PAEMA_{70}$	0.60	0.2386	2.276	72 μL	52 μL
	$PPEGMA_{12}$-$PAEMA_{100}$	0.60	0.1670	2.314	50 μL	36 μL
	$PPEGMA_{12}$-$PAEMA_{125}$	1.50	0.3340	5.826	100 μL	74 μL
	$PPEGMA_{12}$-$PAEMA_{150}$	1.00	0.1856	3.902	56 μL	42 μL
25	$PPEGMA_{12}$-$PAEMA_{70}$	1.00	0.3977	2.792	120 μL	88 μL
	$PPEGMA_{12}$-$PAEMA_{100}$	1.20	0.3340	3.426	100 μL	74 μL
	$PPEGMA_{12}$-$PAEMA_{125}$	1.50	0.3340	4.326	100 μL	74 μL
	$PPEGMA_{12}$-$PAEMA_{150}$	1.50	0.2784	4.354	84 μL	62 μL
20	$PPEGMA_{21}$-$PAEMA_{150}$	1.00	0.3186	3.902	56 μL	42 μL
	$PPEGMA_{21}$-$PAEMA_{200}$	1.00	0.2390	3.928	42 μL	30 μL
	$PPEGMA_{21}$-$PAEMA_{300}$	1.50	0.2390	5.928	42 μL	30 μL

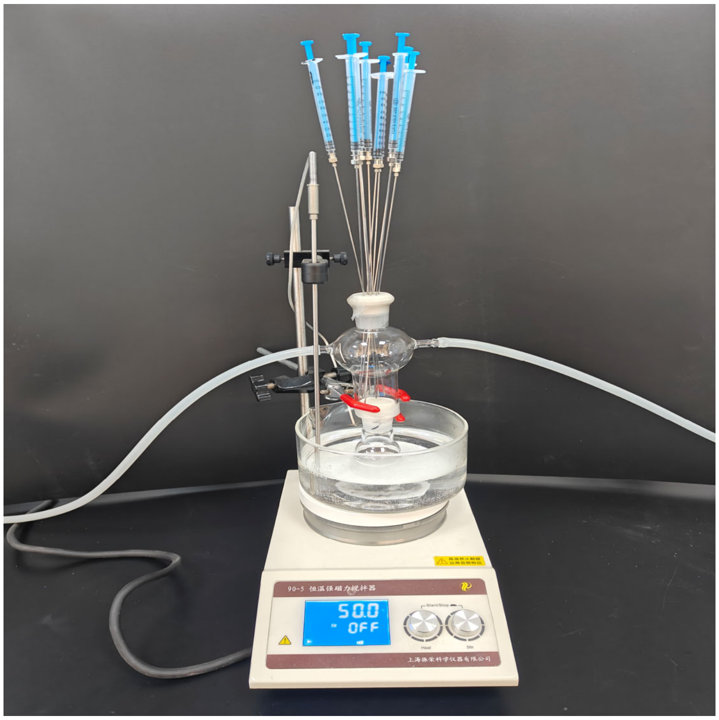

Figure S1. Schematic diagram of the custom-built device for the redox-initiated RAFT emulsion polymerization kinetics of AEMA (nitrogen flow through the tube during operation).

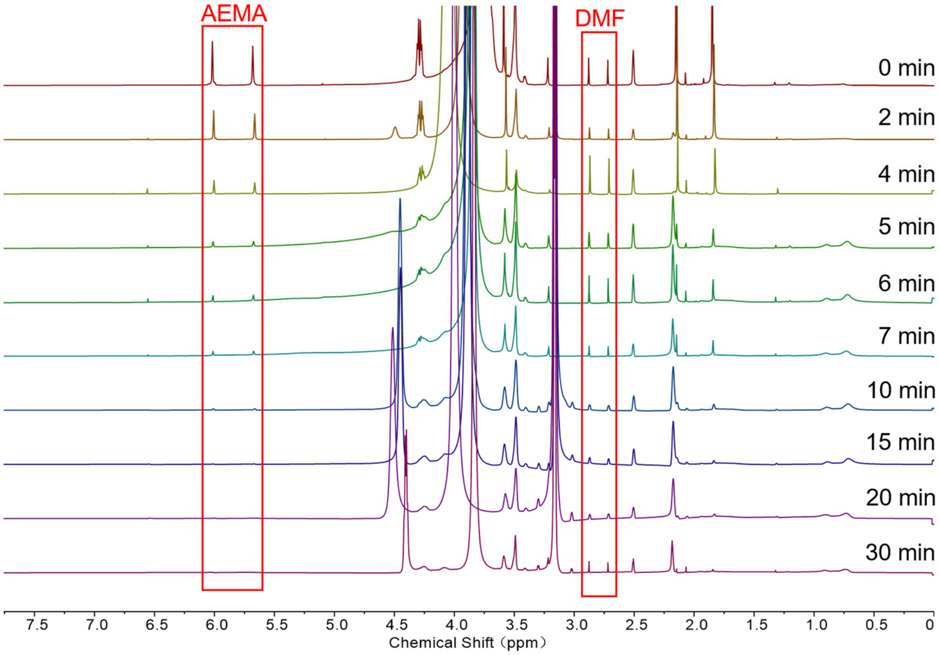

Figure S2. ^{1}H NMR spectra of aliquots withdrawn during RAFT emulsion polymerization of AEMA of 15% w/w, target composition of $PPEGMA_{12}$-$PAEMA_{100}$ at 50 °C. A small amount of DMF was used as the internal standard.

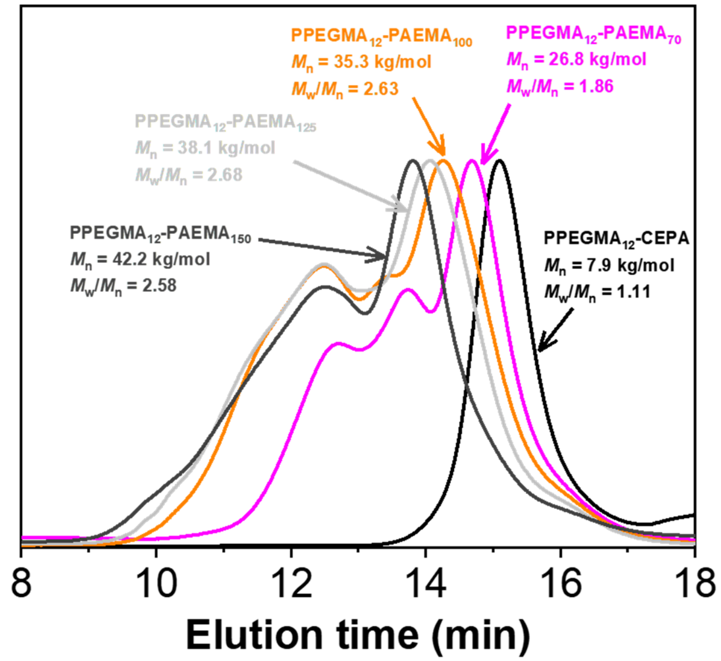

Figure S3. THF GPC traces of diblock copolymers formed by RAFT emulsion polymerization of AEMA (20% w/w).

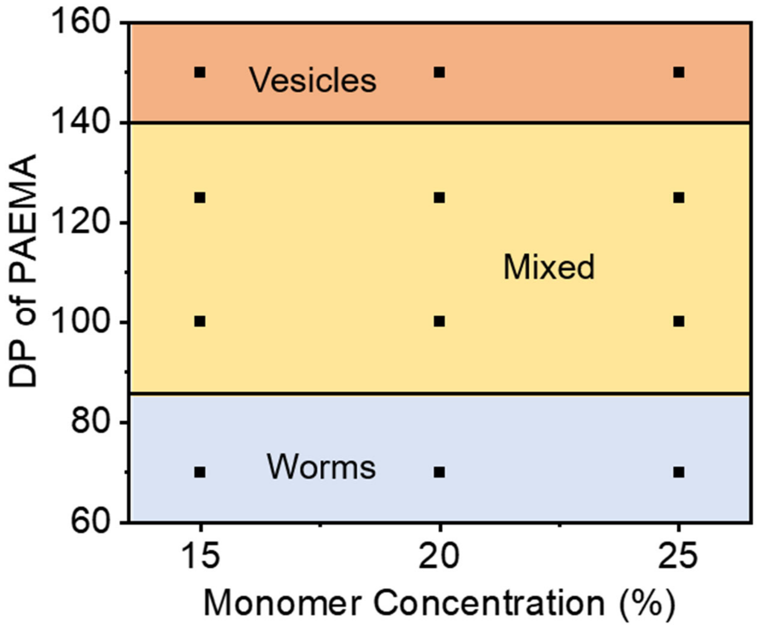

Figure S4. Morphological phase diagram constructed for $PPEGMA_{12}$-$PAEMA_m$ diblock copolymer assemblies prepared by RAFT emulsion polymerization of AEMA.

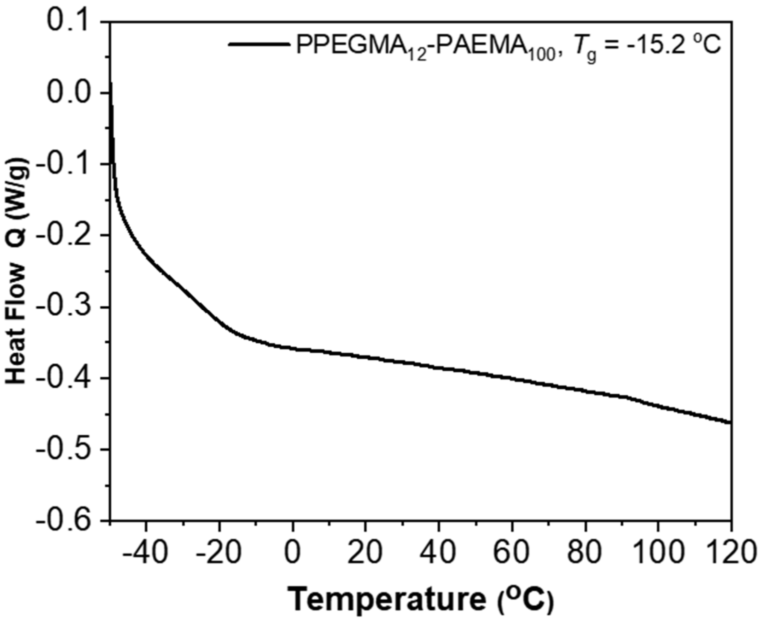

Figure S5. DSC curve of the block copolymer PPEGMA$_{12}$-PAEMA$_{100}$ obtained by RAFT emulsion polymerization of AEMA.

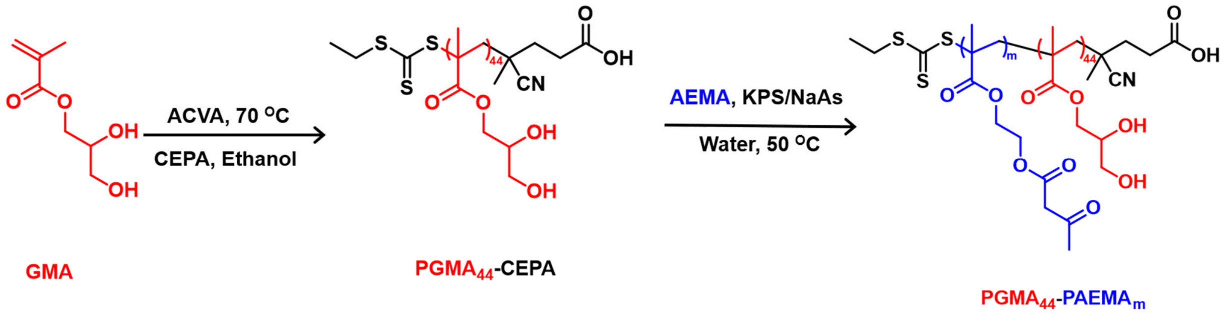

Figure S6. Synthesis of poly(glycerol monomethacrylate)-poly(2-(acetoacetoxy) ethyl methacrylate) (PGMA$_{44}$-PAEMA$_{m}$) diblock copolymer assemblies via redox-initiated RAFT-mediated emulsion polymerization.

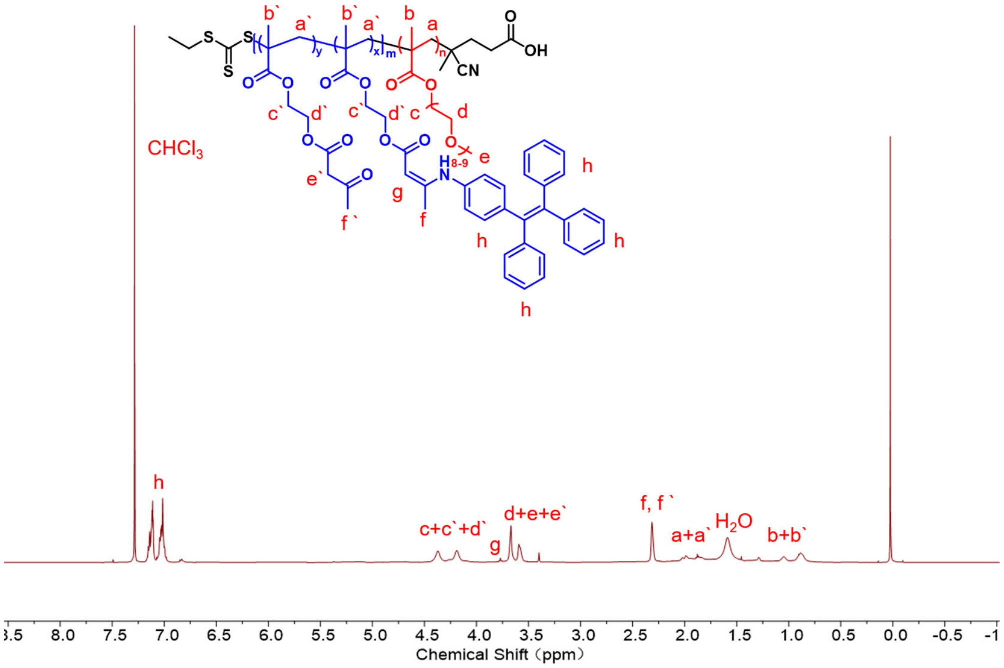

Figure S7. ^{1}H NMR spectra of AIE-polymer assemblies in $CDCl_3$, prepared via post-polymerization modification.

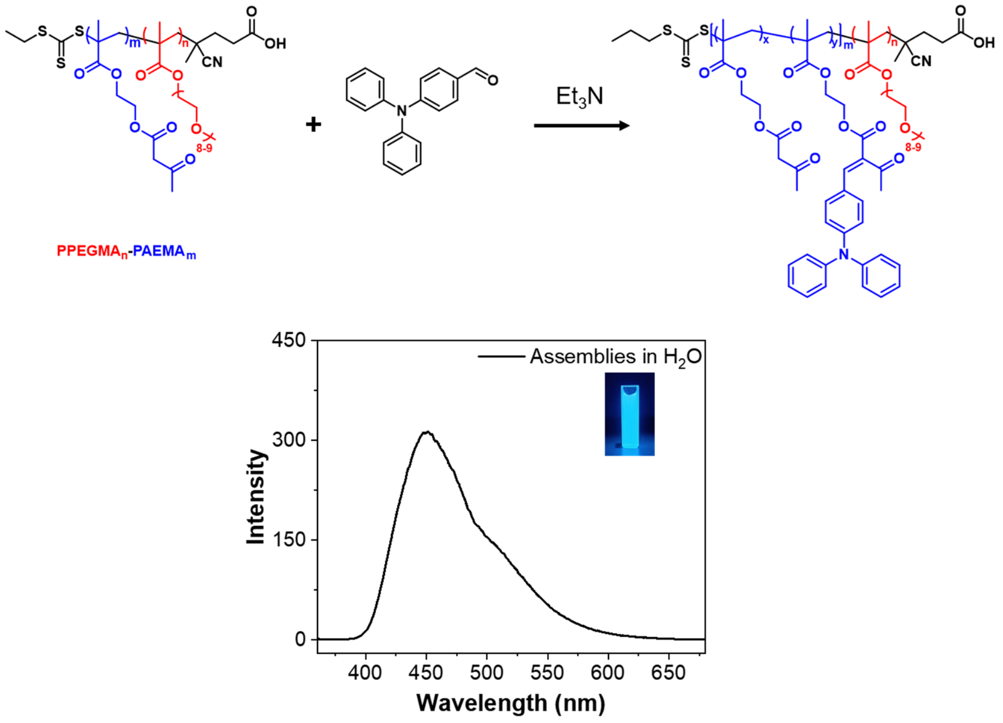

Figure S8. Schematic illustration of the preparation of AIE polymer assemblies via post-polymerization modification with aldehyde-based AIE molecules, along with the fluorescence emission spectra of the AIE polymer assemblies.
